# The incidence of fractures in children under two years of age: a *systematic review*

**DOI:** 10.1186/s12891-024-07633-5

**Published:** 2024-07-09

**Authors:** Karen Rosendahl, Laura Tanturri de Horatio, Celine Habre, Susan C. Shelmerdine, Janina Patsch, Ola Kvist, Regina K. Lein, Domen Plut, Edvard J. Enoksen, Rien Avenarius, Lene B. Laborie, Thomas A. Augdal, Paolo Simoni, Rick R. van Rijn, Amaka C. Offiah, on behalf of the European Society of Paediatric Radiology (ESPR) Musculoskeletal and Child Abuse Task Forces

**Affiliations:** 1 Department of Clinical Medicine, UiT the Artic University of Norway, Tromsø, Norway; 2 Department of Radiology, University Hospital of North Norway, Pb 100, Tromsø, 9038 Norway; 3 Department of Imaging, IRCCS Bambino Gesù Children‘s Hospital, Rome, Italy; 4 Pediatric Radiology Unit, Radiology Division, Diagnostic Department, University Hospitals of Geneva, Geneva, Switzerland; 5 Department of Clinical Radiology, Great Ormond Street Hospital for Children NHS Foundation Trust, Great Ormond Street, London, England; 6 Great Ormond Street Hospital for Children, UCL Great Ormond Street Institute of Child Health, London, England; 7 NIHR Great Ormond Street Hospital Biomedical Research Centre, Bloomsbury, London, England; 8 Department of Radiology, St. George’s Hospital, London, England; 9 Division of General and Pediatric Radiology, Department of Biomedical Imaging and Image-Guided Therapy, Medical University of Vienna, Wien, Austria; 10 Department of Pediatric Radiology, Karolinska University Hospital, Stockholm, Sweden; 11 Department of Women’s and Children’s Health, Karolinska Institute, Solna, Sweden; 12 University Library, Bergen University, Bergen, Norway; 13 Clinical Radiology Institute, University Medical Centre Ljubljana, Ljubljana, Slovenia; 14 Faculty of Medicine, University of Ljubljana, Ljubljana, Slovenia; 15 Section for Paediatric Radiology, Department of Radiology, Haukeland University Hospital, Bergen, Norway; 16 Department of Clinical Medicine, University of Bergen, Bergen, Norway; 17 Department of Radiology, “Reine Fabiola” Children’s University Hospital Université Libre de Bruxelles, Brussels, Belgium; 18 Department of Radiology and Nuclear Medicine, UMC, University of Amsterdam, Amsterdam, Netherlands; 19 Department of Oncology & Metabolism, University of Sheffield, Sheffield, England

**Keywords:** Fracture, Infant, Children, Incidence, Paediatric

## Abstract

**Background:**

Epidemiological research on fractures in children under the age of two is of great importance to help understand differences between accidental and abusive trauma.

**Objective:**

This systematic review aimed to evaluate studies reporting on the incidence of fractures in children under two years of age, excluding birth injuries. Secondary outcome measures included fracture location, mechanisms of injury and fracture characteristics.

**Methods:**

A systematic literature review (1946 to February 7th 2024), including prospective and retrospective cohort studies and cross-sectional cohort studies, was performed. Studies including children from other age groups were included if the actual measures for those aged 0–2 years could be extracted. We also included studies restricted to infants. Annual incidence rates of fractures were extracted and reported as the main result. Critical appraisal of was performed using the Appraisal tool for Cross-Sectional Studies.

**Results:**

Twelve moderate to good quality studies met eligibility criteria, of which seven were based on data from medical records and five were registry studies. Studies investigated different aspects of fractures, making comprehensive synthesis challenging. There was an overall annual fracture incidence rate of 5.3 to 9.5 per 1,000 children from 0–2 years of age; with commonest sites being the radius/ulna (25.2–40.0%), followed by tibia/fibula (17.3–27.6%) and the clavicle (14.6–14.8%) (location based on 3 studies with a total of 407 patients). In infants, the reported incidence ranged between 0.7 to 4.6 per 1,000 (based on 3 studies), with involvement of the clavicle in 22.2% and the distal humerus in 22.2% of cases (based on 1 study). Only a single metaphyseal lesion was reported (proximal humerus of an 11-month-old infant). Fracture mechanisms were detailed in four studies, with fall from chair, bed, table, own height or fall following indoor activities causing 50–60% of fractures.

**Conclusions:**

There is a paucity of good quality data on fracture incidence in children under the age of two. Larger, prospective and unbiased studies would be helpful in determining normal pattern of injuries, so that differences from abusive trauma may be better understood.

**Supplementary Information:**

The online version contains supplementary material available at 10.1186/s12891-024-07633-5.

## Introduction

In children under the age of two, fractures are rare, particularly in non-ambulatory infants, with a predilection for the clavicle and skull in those under 8 months of age [1]. In toddlers between 9 and 24 months of age, forearm and lower leg fractures predominate [1, 2]. The incidence and pattern of fractures in children under the age of two is, however, poorly described in the literature. This age group is particularly vulnerable to inflicted injury, which may be difficult to detect. Both under- and overdiagnosis occur, in part due to limited knowledge of variations in normal growth that may mimic pathology [3–5], limited experience, and subtlety of fractures of immature bone. Although the Royal College of Paediatrics and Child Health website provides important knowledge for those dealing with potential abusive trauma (https://childprotection.rcpch.ac.uk/child-protection-evidence/fractures-systematic-review/), it mainly focuses on fractures indicative of abuse, fracture dating, and rib fractures secondary to cardiopulmonary resuscitation.

In this novel systematic review, we aim to identify and summarise all epidemiological studies which have reported on the incidence of fractures in children under the age of two. Secondary outcome measures include fracture location, mechanisms of injury and fracture characteristics.

## Methods

A systematic literature review was performed in accordance with the Preferred Reporting Items for Systematic Reviews and Meta-Analyses guidelines [6]. The protocol was registered in the International prospective register of systematic reviews (PROSPERO reg. number CRD42022355938). Ethical approval was not required for this review of publicly available data.

### Eligibility criteria

The review includes all studies which have attempted to quantitatively assess the incidence of fractures in children under two years of age; thus, the outcome of interest was the annual incidence rates, with secondary measures being localization, fracture characteristics and mechanisms.

Inclusion criteria applied to identified studies were epidemiological studies, written in English, which attempt to quantitatively assess the incidence of fractures in children under two years of age, including prospective and retrospective cohort studies and cross-sectional cohort studies. Studies including children from other age groups were included if the actual measures for those aged 0–2 years could be extracted. We also included studies restricted to infants (0–1-year-olds).

Excluded were non-primary research, systematic literature reviews, animal studies, in-vitro studies, interventional studies, single case reports, editorials/comments, and clinical guidelines; studies lacking full text or relevant data on outcomes, studies addressing birth injuries alone, studies addressing child abuse alone, studies of children with underlying disease (e.g., osteogenesis imperfecta, leukaemia, metabolic bone disease etc.) and studies restricted to sites other than the limbs or ribs. When studies reported findings from the same population, we selected only the most relevant study based on date, sample size, and reported analysis of data.

### Information sources and search strategy

We comprehensively searched Medline (Ovid), Embase (Ovid), the Cochrane Library, Cinahl (Ebco) and Web-of-Science (Clarivate) for full text articles published in English between 1946 and 7th of February 2024 (RKL/KR, the latter with 35 years of experience in paediatric radiology). Both subject headings and free text words were used for the following concepts: bone fracture, incidence, and children under 2 years of age (detailed search strategies are listed in Additional file 1). We also searched the reference lists of the included articles.

### Screening, study selection and data extraction

Search results were exported through EndNote, version 20 (Clarivate, Philadelphia, US) duplicates were removed, and all eligible studies were imported to Rayyan [7]. Titles and abstracts were screened by one investigator (KR) for possible inclusion according to the pre-specified eligibility criteria [8, 9]. A random sample of 35% of titles and abstracts were double screened by one of two investigators (SCS/LTdH) to ensure high levels of agreement. Any article which the investigator was unsure about was included in the list of full text articles to be reviewed in a second stage. Full text articles were retrieved and assessed for final eligibility by one investigator (KR), and if doubt, in consensus with a second reviewer (RRvR). From the included studies, two reviewers (KR/RRvR) independently extracted relevant data and populated a project-specific Microsoft Excel spreadsheet. Discrepancies between values were discussed and resolved between the reviewers and/or by involving a third reviewer (TAA). The following data were collected: study details (first author, publication year and country), recruitment setting (sample description/hospital/year), study design, sample size (number of children under two years of age/number of fractures), sex and outcome measures (annual incidence rates (per 1,000), location (five most common fracture sites as reported in each paper), mechanism, fracture type (transverse, spiral etc.) and whether the fracture was acute or healing.

### Strategy for data synthesis

The data synthesis was through a narrative analysis method of incidence. Annual incidence rates of fractures (per 1,000) were extracted, and reported as the main result (in total, and by sex / location / mechanism).

### Assessment of methodological quality

Critical appraisal was performed independently by three reviewers (OK, CH, JP, with 7, 5 and 10 years of experience in paediatric radiology, respectively) to assess the quality of included studies and provide context for the interpretation of the findings. Each of the selected studies was evaluated with the Appraisal tool for Cross-Sectional Studies (AXIS) (Additional file 2), focusing on the presented aims, methods and analysis of what is reported [10]. As the tool does not provide a numerical scale for assessing the quality of a study, a degree of subjectivity was used to classify the studies into poor, fair, moderate or good quality [10]. When studies included multiple analyses aimed at answering several research questions within the same study, quality assessments were only applied to the analyses relevant to this systematic review.

## Results

**Fig. 1:**
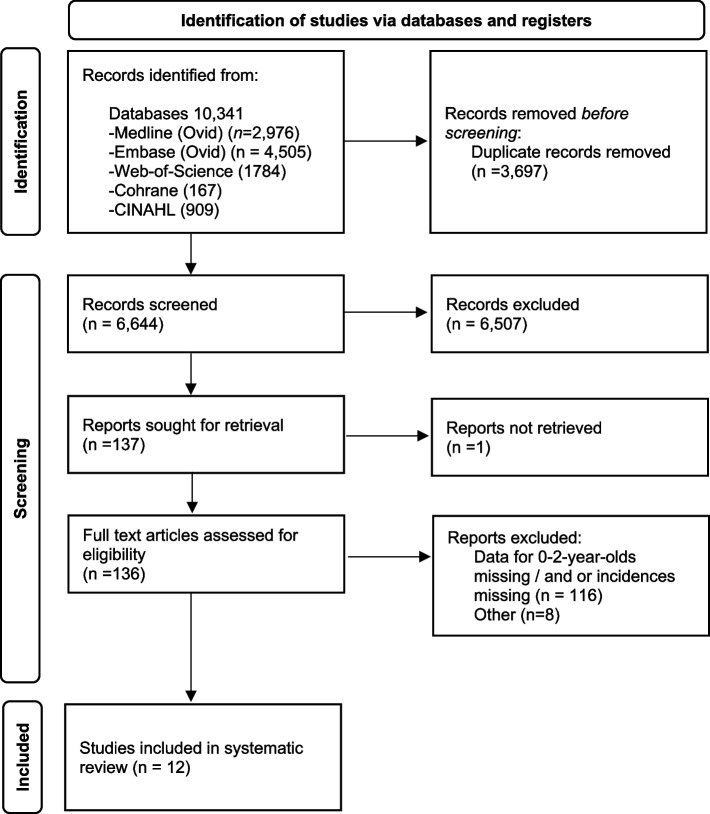
PRISMA flow diagram outlining the process by which articles were screened

A total of 10,341 references were found following the literature search (Fig. 1). After removal of duplicates, 6,644 titles/abstracts were screened for relevance, of which 6,507 were excluded. After a full-text review of the remaining 136 studies, 12 were eventually included (Fig. 1).

### Characteristics of studies

Of the 12 included studies, 7 were based on review of medical records; of which 5 were single hospital studies [1, 2, 11–13], 1 was based on medical records from two paediatric trauma units [12] and 1 on data from 27 hospitals and 126 clinics [14]. Five were registry studies [15–19].

Two were prospective [12, 13], 5 were retrospective cohort studies [1, 2, 11, 14, 20], and 5 were retrospective registry studies. In 3 of the studies, all radiographs were re-assessed by a radiologist or by an orthopaedic surgeon to minimize misdiagnosis [2, 13, 20] (Table 1).

**Table 1:** Characteristics of included studies: setting, study design and size, annual fracture incidences and quality score according to AXIS

1st author, year, country	Recruitment setting (sample description / hospital / year)	Study design	Number of children < 2 years of age / fractures (% male)	Annual incidence rates per 1,000 (% male)	Quality
Tiderius CL 1999, Sweden [11]	All children < 16 years seen at the only ED in Malmø /1993–1994. Fractures to the ribs and teeth excluded	Retrospective cohort study, with review of uncertain radiographs	Not given	9.5 per 1,000 (Fig. 1)	Good
Clarke N 2012, England/UK [1]	All children < 2 years seen at one ED in Southampton, with fracture(s) / 2007–2008. Birth injury excluded	Retrospective cohort study	3370 / 123 (37.4)	5.3 per 1,000	Good
Rosendahl K 2021, Norway [2]	All children < 2 years of age seen at the only AE department in Bergen, due to trauma warranting a radiograph / 2010–2015. Birth injury and high energy trauma excluded	Retrospective cohort study, with paediatric radiologist. single-examiner review of all radiographs	408 / 162 (49.9)	5.4 per 1,000 (0.7/1000 < 1 year of age increasing to 7.3 per 1,000 from 1–2-year-olds)	Good
Hansoti B 2008, Scotland/UK [12]	All children < 2 years seen at one emergency department (ED) in Edinburgh, with fracture (s) / 2003. Birth injury excluded	Prospective database, with retrospective retrieval of data and review of some radiographs ***Limb fractures only***	122 / 122 (not given)	Limb fractures 4.6 per 1000 < 1 year of age, increasing to 17 per 1,000 from 1–2-year-olds	Moderate
Mamoowala NA 2019, England/UK [13]	All children < 16 years of age presented to the A&ED in Leicester with a radiographically confirmed fracture of the distal radius / 2007–2014. Birth injury excluded	Prospective study, with orthopedic review of XRs ***Distal radius only***	245 / 245 (46.9)	Distal radius 0.8 per 1,000	Moderate
Talbot C 2018, England/UK [15]	All children < 15 years of age registered in TARN / 2012–2015 with a closed fracture to the femur shaft	Trauma Audit Research Network (TARN) data ***Femur shaft only***	Not given	Femur shaft 0.07 per 1000 < 1 year of age, increasing to 12.1 per 1,000 for 1–2-year-olds	Moderate
Hinton RY 1999, US [16]	All children < 18 years of age / 1990–1996 Data from the United States Bureau of the Census for the state of Maryland for the year 1990 were used to obtain denominator data	Hospital discharge Database of the Maryland Health Services Cost Review Commission for the years 1990- 1997 ***Femur only***	Not given / 238 (not given)	Femur 0.3 per 1,000	Moderate
Bridgman S 2004, England/UK [17]	All children < 15 years admitted to hospital in the WEST Midlands of England / 1991–2002 Population estimates for 1991 to 2001 based on the 1991 national census, were used as denominators to calculate annual incidence rates	NHS Hospital Episode Statistics (HES) collected by the Department of Health ***Femur only***	Not given	Femur 0.5 per 1,000 (50) (Fig. 1)	Moderate
Rennie L 2007, Scotland/UK [20]	All children < 16 years seen at two pediatric trauma units in Edinburgh, with fracture(s) / 2000. Birth injuries, skull and rib fractures excluded	Retrospective cohort study, with single-examiner review of all XRs	Not given	3.6 per 1000 (47%) < 1 year of age	Good
Powell EC 2002, US [19]	All children < 1 year of age registered during 1992–1999	ED survey from the National Center for Health Statistics National Hospital Ambulatory Medical Care Survey. ***Extremity fractures only***	Not given	Extremity fractures 4.6 per 1,000 < **1 year of age**	Moderate
Heideken J 2011, Sweden [18]	All children < 14 years of age admitted for fracture to the femur shaft during 1987–2005. Birth injury excluded	Data collected from the Swedish National Hospital Discharge Registry (SNHDR) ***Femur shaft only***	313 < 1 year of age / 49% male	Femur shaft 0.2 per 1,000 (m/f = 0.9) < **1 year of age**	Moderate
Hagino H 2000, Japan [14]	All children < 20 years presenting to 23/27 hospitals and 121/126 clinics in Tottori Prefecture / 1992–1995	Retrospective cohort study ***Distal radius only***	Not given	Distal radius 0.2 per 1,000 for both sexes < **1 year of age**	Moderate

*AE* Accident and Emergency, *AXIS* Appraisal tool for Cross-sectional Studies, *ED* Emergency Department

All 12 studies were performed in, or using data from cities and/or rural areas; 4 studies in the UK [1, 13, 15, 17], 2 in the US [16, 19], 2 in Scotland [12, 20], 2 in Sweden [11, 18], 1 in Norway [2], and 1 in Japan [14]. Sample size was given for 5 out of 8 studies on children < 2 years of age (mean 178 fractures, range 122–245) and for 1 of 4 studies including infants (Table 1).

Four studies included all relevant fracture locations [1, 2, 11, 20], while the remainder reported on the incidence of fractures to the appendicular skeleton [12, 19], to the femur [15–18] or to the distal radius [13, 14] (Table 1).

All studies were considered of moderate to good quality based on the AXIS system, although several were lacking population denominator and census-based demographic data necessary to generate true incidence rates (Table 1). Study design limitations were mainly due to potential selection bias or unadjusted confounders. Important potential confounders, such as socioeconomic status or additional comorbidities were not accounted for in any of the analyses.

### Incidence estimates

Study results are summarized in Tables 1 and 2. The overall annual fracture incidence rates for children under two years of age was reported at 5.3 to 9.5 per 1,000 [1, 2, 11], while the incidence for children under the age of one ranged from 0.7 to 4.6 per 1,000 [2, 12, 20]. The incidence of limb fractures was reported at 4.6 per 1,000 amongst infants, rising to 7.3 per 1,000 for those between one and two years of age [12].

**Table 2:** The five most common fracture sites and mechanisms, when given in studies addressing children 0–2 years and 0–1 years, separately

1st author, year, Country / *n* = number of fractures	5 most common fracture sites, *n* (%)	Mechanisms	Type of fracture, *n* (%)
**0–2 years of age**			
Clarke N, 2012 [1], England/UK / *n* = 123	Tibia/fibula, 34 (27.6) Radius/ulna, 31 (25.2) Clavicle, 18 (14.6) Humerus, 12 (9.8) Femur and skull, 6 (4.9)	Insignificant injury^a,b^: 57.7% 12.2% had unexplained histories with no mechanism identified	Not given
Rosendahl K, 2021 [2], Norway / *n* = 162	Radius, 37 (22.8) Ulna, 18 (11.1) Tibia, 28 (17.3) Clavicle, 24 (14.8) Phalanx hand, 19 (11.7) Classic metaphyseal lesion 1 (0.6)	Fall from chair, bed, table or own height: 60% In 19.5% there was a mismatch between the fracture mechanism given and the findings. CML: pulling arm	Complete (simple, wedge, complex) 68 (42.0) Buckle/ greenstick 52 (32.1) Avulsions 20 (12.3) fissures 11 (6.8)
Hansoti B, 2008 [12], Scotland/UK / *n* = 122	Radius and/or ulna, 49 (40) Tibia and/or fibula, 26 (21) Hand, 16 (13) Humerus, 14 (12) Foot, 8 (7) ***Upper (incl.clavicles) and lower limbs) only***	Fall (height not specified): 52% 5 children referred for child-protection review since no convincing cause could be found for the injury	Buckle / greenstick of the radius and ulna, 38 (31.2) transverse/oblique/ spiral of metaphysis, 20 (16.4)
Talbot C, 2018 [15], England/UK / *n* = not given	***Femur fractures only***	Fall < 2 m in the majority, around 70%	Not given
Hinton RY, 1999 [16], US / *n* = 238	***Femur fractures only***	Falls 63% Abuse 14% Unspecified 12% Motor vehicle accident 4% Struck 4% Other 3% (Figures deduced from a bar graph [Fig. 3] in [12])	Not given
Bridgman S, 2004 [17], England/UK / *n* = not given	***Femur fractures only***	Falls were recorded as the cause in 76.7% of one-year-olds Maltreatment was recorded as the external cause in 7.8% of children aged less than one year	Not given
**0–1 year of age**			
Rennie L, 2007 [20], Scotland/UK / *n* = not given	Clavicle (22.2%) Distal humerus (22.2%) Distal radius (11.1%) Radius/ulna diaphysis (11.1%) Tibia/fibula (8.9%)		Not given
Heideken J, 2011 [18], SE	***Femur fractures only***	External causes: -fall < 1 m: 5.4% / 4.5% m/f -fall > 1 m: 2.6% / 4.2% m/f -fall unspecified 3.8% / 2.6% m/f	Not given

^a^Falls from sofas/beds and indoor activities

^b^4 children had an additional intracranial haemorrhage

Femur fractures had an incidence rate range of 0.07–0.2 per 1,000 for infants [15, 18], increasing to 0.3–0.5 per 1,000 for 0–2-year-olds [16, 17]. For 1–2-year-olds, the corresponding figure was 12.1 per 1,000 [15].

Three studies reported on sex distribution, of which two found fractures to be equally distributed between sexes; one addressing all except high energy traumas fractures in children 0–2 years of age [2] and the other addressing fractures to the distal radius in infants [14]. The third study reported on more fractures in girls than in boys; 62.6% vs 37.4% [1].

### Most common fracture locations

Three papers reported on the most common fracture sites; in 0–2-year-olds the radius/ulna (25.2–40% of all fractures), followed by the tibia/fibula (17.3–27.6%), and the clavicle (14.6–14.8%) [1, 2, 12] (Table 2). In infants, the most common fracture sites were the clavicle and distal humerus (22.2% each of all fractures) [20].

### Fracture mechanisms

Fracture mechanisms were reported in 7 studies, of which 2 of the 3 studies including all locations in 0–2-year-olds, described fall from low height (chair, bed, table, own height) to cause 50–70% of fractures [1, 2], while a third study described fall, without specifying height, as the cause in 52% [12] (Table 2). Five studies reported on abuse as a potential mechanism in 4.1%—12.2% of the cases [1, 2, 12, 16, 17]. As for fractures to the femur, falls were the reported mechanism in 24–77% of the cases [15–18], of which two studies specified the height [15, 18]. In the Swedish registry study from 2011 including 313 infants with femur fractures, birth injuries excluded, the authors found that 70 (22.4%) out of 313 fractures were caused by a fall, of which 31 from a height < 1 m, 19 from a height > 1 m, whilst the remainder 20 were unspecified [18]. In the study from Talbot et al., the most common mechanism was fall of less than two meters [15].

### Type of fractures

Two studies reported on fracture type [2, 12], 31–32% being of the buckle/greenstick type (Table 2). Only a single classical metaphyseal lesion (CML) (in a proximal humerus of an 11-month-old infant) was reported [2]. The fracture was initially missed, but diagnosed during the retrospective review of the radiographs. The child refused to use the arm, however, there was no mention of trauma in the medical notes.

### Acute/healing fracture

The incidence of healing fractures was reported at 0.3 per 1,000 in children under two years of age [2]. This information could not be extracted for those under 1 year of age.

## Discussion

The purpose of this review was to systematically investigate the existing literature to determine the population-based fracture incidence in children under the age of two years. Although there was a vast body of literature reporting fractures in children, most papers did not report figures for 0–2-year-olds specifically. Moreover, studies were lacking the appropriate population denominator and census-based demographic data necessary to generate true incidence rates rather than frequencies or proportions. Studies differed in design; methods to secure a population-based cohort; type of health service where the study was undertaken; and clinical setting. The degree of variation across the studies, combined with our quality findings that most studies were at risk of bias, meant that it was not appropriate to pool the results in a meta-analysis.

Most included studies were based on researcher-collected data from medical records, while five were registry based. Despite the increasing use, no developed methodological literature on use and evaluation of population based registers is available [21]. Although complete study populations minimize selection bias, registry studies are limited by missing data, lack of data quality, confounder information, and the risk of data dredging. On the other hand, data retrospectively collected from medical records suffer similar limitations, underscoring the need for prospective studies and validated research databases.

Knowledge of fractures in children has typically come from Northern European population studies reported in the late 1970s through the 80 s and 90 s [22–25], however, most of these have provided pooled data from birth until school-age or until skeletal maturity without focusing on the youngest age group. Despite performing an extensive literature search, we identified only three studies reporting true, population-based incidences in 0–2-year-olds [1, 2, 11]. The reported incidences were relatively similar, ranging from 5.3 to 9.5 per 1,000, of which the latter comes with a caveat being deduced from a figure in the original paper.

Three studies addressed infants, with reported fracture incidences ranging from 0.7 per 1,000 in a Norwegian study [2] to 3.6 and 4.6 per 1,000 in two studies from Scotland [12, 20]. The differences may in part be due to selection bias, as one study excluded high energy trauma [2], another excluded skull and rib fractures [20] and a third excluded the axial skeleton, pelvis and chest, but included the clavicles [12]. The many different methodological and reporting approaches highlights the challenges of synthesising results. Although there was high heterogeneity of the studies included, two studies concurred and showed significantly higher fracture rates in 1–2-year-olds compared to infants [2, 12]. This finding seems reasonable, as fractures are less likely to occur in non-ambulatory infants.

Our review found that fractures to the forearm constituted up to 50% of all fractures in children aged 0–2 years, as compared to around 20% in infants. However, the number of studies is low, reducing confidence in this finding. Interestingly, only a single CML (of the proximal humerus of an 11-month-old infant) was reported despite the thousands reviewed. The child was brought to the emergency out-patient clinic because he refused to use his left arm, with no history of trauma. Unfortunately, the fracture was missed during the initial visit, thus the finding did not trigger a more extensive work-up. In retrospect, the authors speculate that the fracture might have represented a missed, inflicted injury [2, 3].

In terms of fracture mechanisms, insignificant injury or fall from low height such as chair, bed, table or own height, was the reported mechanism in 50–60% of all fractures amongst 0–2-year-olds, while this was the case for one tenth of femur fractures in infants. However, these results must be interpreted with care, as none of the studies registered fracture mechanisms in a detailed, prospective manner. Moreover, a significant proportion of the injuries were not observed by the caretakers or by other adults, thus, the figures given include potentially abusive fractures. However, it was not the purpose of this review to examine the incidence of inflicted injury.

The strengths of this systematic review include the rigorous methodological approach employed using an established methodological framework. A comprehensive search strategy was used, with broad inclusion criteria. Three independent reviewers were involved in the screening process to identify papers for full-text reading, and a fourth reviewer was included in data extraction. Moreover, the search was repeated at the time of manuscript preparation to capture recent and relevant studies.

There are some limitations to the present study. First, the number of studies was low with varying quality, and many did not report essential data, such as incidences by sex. Second, given the limitations of the reported data, the risk of bias among the included studies and the wide heterogeneity between them, we were unable to combine data in a meta-analysis, and instead results were reported as a narrative summary. Thirdly, we included articles written in English only. We also planned to assess publication bias but were unable to do so owing to the wide heterogeneity between the included studies. The generalisability of these findings may be uncertain.

## Conclusion

There is a paucity of good quality data on fracture incidence in children under the age of two. This systematic review of the literature found only 12 studies over the last 78 years that met the eligibility criteria, however, due to data inhomogeneity a meta-analysis could not be calculated. From the limited, potentially biased data available, we calculated the following: an overall incidence of fractures of around 1% in children under 2-year-olds, most of which were lower leg or forearm fractures, and a lower incidence in infants (under 1-year-olds) being a maximum of 0.5%, most of which were clavicle and humeral fractures. The low frequency of CMLs and absence of rib fractures may be differentiating features from inflicted injury.

### Supplementary Information

Supplementary Material 1.

Supplementary Material 2.

## Data Availability

The datasets used and/or analysed during the current study are available from the corresponding author on reasonable request.
