# Supplementary material for: The incidence of fractures in children under two years of age: a systematic review
Source: BMC Musculoskelet Disord. 2024 Jul 9;25:528. doi: 10.1186/s12891-024-07633-5 (PMC11232341; doi:10.1186/s12891-024-07633-5)
Supplement: Supplementary file 1 — Supplementary Material 1. [file 12891_2024_7633_MOESM1_ESM.docx]

Search strategies: Februar 2024

**Ovid MEDLINE(R) and Epub Ahead of Print, In-Process, In-Data-Review & Other Non-Indexed Citations and Daily <1946 to February 06, 2024>; Search Date 7 Feb 2024**

1 exp Fractures, Bone/ 212578

2 (fracture* or broken bone* or "break* in bone*" or classic* metaphyseal lesion* or CML).ti,ab,kf. 341207

3 1 or 2 383867

4 incidence/ 304666

5 (Incidence* or positivity rate*).ti,ab,kf. 972201

6 4 or 5 1083561

7 exp Infant/ 1267097

8 (Newborn or Newborns or New-born or New-borns or Baby or Babies or Infant or Infants or Infanc* or Toddler* or Pre-school* or preschool* or "pre school*" or pediatri* or paediatr* or neonat* or suckling or boy or boys or girl or girls or kindergar* or kid or kids or "early childhood").ti,ab,kf. 1603432

9 7 or 8 2195891

10 3 and 6 and 9 2976

<https://ovidsp.ovid.com/ovidweb.cgi?T=JS&NEWS=N&PAGE=main&SHAREDSEARCHID=3wWDaU0JU6Zluq67e0MW2AHacqZbRXXqpsXIP75jI64r8ugdqfuQZP6ZLJdzUYDa>

Comments on Ovid-databases:
/ = search on subject heading

Exp = search on a subject heading, expanded to include narrower terms

Ti,ab,kf = search in title, abstract and authors keywords of the article

**Embase (Ovid) <1974 to 2024 February 06>; Search Date 7 Feb 2024**

1 exp fracture/ 362339

2 (fracture* or broken bone* or "break* in bone*" or classic* metaphyseal lesion* or CML).ti,ab,kf. 413019

3 1 or 2 506787

4 exp incidence/ 703373

5 (Incidence* or positivity rate*).ti,ab,kf. 1412321

6 4 or 5 1596311

7 exp infant/ 1158657

8 exp toddler/ or exp preschool child/ 638047

9 (Newborn or Newborns or New-born or New-borns or Baby or Babies or Infant or Infants or Infanc* or Toddler* or Pre-school* or preschool* or "pre school*" or pediatri* or paediatr* or neonat* or suckling or boy or boys or girl or girls or kindergar* or kid or kids or "early childhood").ti,ab,kf. 2057041

10 7 or 8 or 9 2733195

11 3 and 6 and 10 4505

<https://ovidsp.ovid.com/ovidweb.cgi?T=JS&NEWS=N&PAGE=main&SHAREDSEARCHID=68RNsIyXZC8AKzjQY25HSa4SAcp76o7Yt07YkPzrj2ykfkNCj136G6bxB3DhQck8W>

**Web of Science (Clarivate), covering WOS.SCI: 1945 to 2024, WOS.AHCI: 1975 to 2024, WOS.ESCI: 2019 to 2024, WOS.SSCI: 1956 to 2024. Search Date 7 Feb 2024**

1: fracture* or "broken bone*" or "break* in bone*" or "classic* metaphyseal lesion*" or CML (Topic) Results: 612053

2: Incidence* or "positivity rate*" (Topic) Results: 927008

3: **Newborn or Newborns or New-born or New-borns or Baby or Babies or Infant or Infants or Infanc* or Toddler* or Pre-school* or Preschool* or (Pre school*) or pediatri* or paediatr* or neonat* or suckling or boy or boys or girl or girls or kindergar* or kid or kids or "early childhood"** (Topic) Results: 1,736,555

4: #1 AND #2 AND #3 Results: 1784

**Cochrane Library (Wiley); Search Date 7 Feb 2024**

# #1 (fracture* or (broken NEXT bone*) or (break* NEAR/2 bone*) or (classic* NEXT "metaphyseal lesion") or (classic* NEXT "metaphyseal lesions") or CML):ti,ab,kw 31525

# #2 (Incidence* or (positivity NEXT rate*)):ti,ab,kw 154514

# #3 (Newborn or Newborns or New-born or New-borns or Baby or Babies or Infant or Infants or Infanc* or Toddler* or Pre-school* or preschool* or (pre NEXT school*) or pediatri* or paediatr* or neonat* or suckling or boy or boys or girl or girls or kindergar* or kid or kids or "early childhood"):ti,ab,kw 161540

# #4 #1 AND #2 AND #3 168 (6 syst.reviews, 161 trials, 1 editorial) = **167 records**

**CINAHL (Ebsco) 1981-current; Seach date 7 Feb 2024**

S1 (MH "Fractures+") 69,532

S2 TI ( fracture* or "broken bone*" or "break* in bone*" or "classic* metaphyseal lesion*" or CML ) OR AB ( fracture* or "broken bone*" or "break* in bone*" or "classic* metaphyseal lesion*" or CML ) 91,577

S3 S1 OR S2 106,051

S4 (MH "Incidence") 81,935

S5 TI ( Incidence* or "positivity rate*" ) OR AB ( Incidence* or "positivity rate*" ) 211,971

S6 S4 OR S5 244,543

S7 TI ( Newborn or Newborns or New-born or New-borns or Baby or Babies or Infant or Infants or Infanc* or Toddler* or Pre-school* or preschool* or "pre school*" or pediatri* or paediatr* or neonat* or suckling or boy or boys or girl or girls or kindergar* or kid or kids or "early childhood" ) OR AB ( Newborn or Newborns or New-born or New-borns or Baby or Babies or Infant or Infants or Infanc* or Toddler* or Pre-school* or preschool* or "pre school*" or pediatri* or paediatr* or neonat* or suckling or boy or boys or girl or girls or kindergar* or kid or kids or "early childhood" ) 485,976

S8 (MH "Infant+") 288,499

S9 S7 OR S8 606,700

S10 S3 AND S6 AND S9 909

# Search strategies are set up and run by Regina Küfner Lein, Medical library, University of Bergen, Norway
