# Supplementary material for: The incidence of fractures in children under two years of age: a systematic review
Source: BMC Musculoskelet Disord. 2024 Jul 9;25:528. doi: 10.1186/s12891-024-07633-5 (PMC11232341; doi:10.1186/s12891-024-07633-5)
Supplement: Supplementary file 2 — Supplementary Material 2. [file 12891_2024_7633_MOESM2_ESM.pdf]

Appraisal of Cross-sectional Studies

	Question	Yes	No	Don't know/ Comment
Introduction				
1	Were the aims/objectives of the study clear?			
Methods				
2	Was the study design appropriate for the stated aim(s)?			
3	Was the sample size justified?			
4	Was the target/reference population clearly defined? (Is it clear who the research was about?)			
5	Was the sample frame taken from an appropriate population base so that it closely represented the target/reference population under investigation?			
6	Was the selection process likely to select subjects/participants that were representative of the target/reference population under investigation?			
7	Were measures undertaken to address and categorise non-responders?			
8	Were the risk factor and outcome variables measured appropriate to the aims of the study?			
9	Were the risk factor and outcome variables measured correctly using instruments/measurements that had been trialled, piloted or published previously?			
10	Is it clear what was used to determined statistical significance and/or precision estimates? (e.g. p-values, confidence intervals)			
11	Were the methods (including statistical methods) sufficiently described to enable them to be repeated?			
Results				
12	Were the basic data adequately described?			
13	Does the response rate raise concerns about non-response bias?			
14	If appropriate, was information about non-responders described?			
15	Were the results internally consistent?			
16	Were the results presented for all the analyses described in the methods?			
Discussion				
17	Were the authors' discussions and conclusions justified by the results?			
18	Were the limitations of the study discussed?			
Other				
19	Were there any funding sources or conflicts of interest that may affect the authors' interpretation of the results?			
20	Was ethical approval or consent of participants attained?			
